# Noninvasive detection of alarming intracranial pressure changes by auditory monitoring in early management of brain injury: a prospective invasive versus noninvasive study

**DOI:** 10.1186/s13054-017-1616-2

**Published:** 2017-02-21

**Authors:** Fabrice Giraudet, François Longeras, Aurélien Mulliez, Aurélie Thalamy, Bruno Pereira, Paul Avan, Laurent Sakka

**Affiliations:** 1 University Clermont Auvergne, Laboratory of Neurosensory Biophysics, UMR INSERM 1107, Clermont-Ferrand, France; 2 Department of Anesthesiology and Intensive Care, University Hospital, rue Montalembert, Clermont-Ferrand, 63000 France; 3 Department of Biostatistics, University Hospital, PO Box 69, Clermont-Ferrand, 63003 France; 4 Department of Clinical Research and Innovation, University Hospital, PO Box 69, Clermont-Ferrand, 63003 France; 5 Centre Jean Perrin, 30 rue Montalembert, Clermont-Ferrand, 63000 France; 6 School of Medicine, 28 Place Henri Dunant, Clermont-Ferrand, 63000 France

**Keywords:** Intracranial pressure, Cochlear electrophysiology, Noninvasive monitoring

## Abstract

**Background:**

In brain-injured patients intracranial pressure (ICP) is monitored invasively by a ventricular or intraparenchymal transducer. The procedure requires specific expertise and exposes the patient to complications such as malposition, hemorrhage or infection. As inner-ear fluid compartments are connected to the cerebrospinal fluid space, ICP changes elicit subtle changes in the physiology of the inner ear. Notably, we previously demonstrated that the phase of cochlear microphonic potential (CM) generated by sound stimuli rotates with ICP. The aim of our study was to validate the monitoring of CM as a noninvasive method to follow ICP.

**Methods:**

Non-invasive measure of CM-phase was compared to ICP recorded invasively in a prospective series of patients with acute brain injury managed in a neuro-intensive care unit. The study focused on patients with varying ICP and normal middle-ear function.

**Results:**

In the 24 patients with less than 4 days of endotracheal ventilation and whose ICP fluctuated (50-hour data), we demonstrated close correlation between CM-phase rotation and ICP (average 1.26 degrees/mmHg). As a binary classifier, CM phase changes of 7–10 degrees signaled 7.5-mmHg ICP increases with a sensitivity of 83% and 19% fallout.

**Conclusion:**

Reference methods to measure ICP require the surgical placement of a pressure transducer. Noninvasive CM-based monitoring of ICP might be beneficial to early management of brain-injured patients with initially preserved consciousness and to the diagnosis of neurological conditions, whenever invasive monitoring cannot be performed.

**Trial registration:**

ClinicalTrials.gov NCT01685476, registered on 30 August 2012.

**Electronic supplementary material:**

The online version of this article (doi:10.1186/s13054-017-1616-2) contains supplementary material, which is available to authorized users.

## Background

In brain-injured patients, intracranial pressure (ICP) is monitored to manage cerebral perfusion pressure and prevent secondary lesions [1, 2]. The gold standard for monitoring ICP is provided by the surgical placement of a ventricular or intraparenchymal transducer [3–5]. These invasive procedures require specific expertise and expose to malposition, hemorrhage and infection. Therefore, they are restricted to critical situations in which cerebral perfusion pressure monitoring is mandatory. Conversely, in intermediate situations such as the diagnosis of migraine, headache, glaucoma and initial management of moderate traumatic brain injury, non-invasive methods that would reliably measure ICP are in great demand.

Cochlear sensory-cell responses to sound stimuli may address this challenge. Their detection is routinely applied in screening of neonatal hearing, by paramedics after minimal training. The sensitivity of cochlear sensory-cell responses to ICP changes results from a balance between cerebrospinal and intralabyrinthine fluid pressures [6–8], such that by modifying the ossicular chain stiffness, ICP variations rotate the phase of cochlear responses. Noninvasive monitoring using either pasted skin electrodes or a microphone in the external acoustic meatus allows ICP follow up [6]. Here, we monitored cochlear microphonics (CM) [9], a sufficiently robust response that circumvents the negative influence of noisy environments and of age-related presbycusis. Concomitant invasive ICP and non-invasive CM recordings were compared over several hours in brain-injured patients, so as to determine whether one can establish a limit in CM-phase change that would signal a potentially dangerous ICP trend.

## Methods

### Experimental design

The objective of the research was to confront noninvasive ICP monitoring using CM with invasive ICP monitoring in patients with acute brain injury managed in a neuro-intensive care unit (NICU). During the one-year enrolment period, 53 consecutive patients were enrolled in this prospective, observational study. The inclusion criterion was the possibility of organizing a monitoring session without disturbing the NICU schedule, and prior to this session, of obtaining informed consent from an authorized person. In view of the collected raw data, all patients measured more than 4 days after NICU referral were excluded from further analysis. The main reason was the lack of sufficient ICP fluctuations in these patients, as the intended search for correlation between CM and ICP is valid only if the independent variable, ICP, exhibits enough variability.

### Patients

The patients had been referred to the NICU for head trauma, including brain hemorrhage, tumors, stroke or meningitis. They were intubated, mechanically ventilated and sedated with sufentanil plus midazolam or remifentanil using target*-*controlled infusions [10].

### CM and ICP recordings

The CM is a voltage that results from sound-induced currents flowing at the frequency of a sound stimulus through the transduction ion channels of auditory sensory cells. Otoacoustic emissions, similarly sensitive to ICP changes [6, 11], are sounds reemitted by the same cells, also as a result of transduction. CM is detected noninvasively between a gold-coated electrode (tip*-*trode™, active) inserted in the external acoustic meatus and skin electrodes pasted on the forehead (passive and ground). The CM-eliciting stimulus is a tone sent by a calibrated earphone (see Additional file 1).

The chosen stimulus frequency, near 1000 Hz, is the frequency most sensitive to ICP changes [7, 9]. The voltage between the electrodes is amplified (×100,000) and digitally converted for on-line spectral analysis. It is made of the CM, frequency-locked to the stimulus, and of components associated with cortical and muscular activity, with a broadband frequency spectrum that falls mainly below 500 Hz. Possible electric artifacts from the NICU are generally made up of lines at integer multiples of the mains frequency, distinct from the stimulus frequency. With a sound stimulus of 80–85 dB sound pressure level (SPL) applied for 10–20 s, the CM is at least 10 dB above background electric noise, which guarantees a valid phase measurement, stable as long as ICP does not change. CM was preferred to otoacoustic emissions for which instrumentally generated artefacts occur when the level of sound-eliciting stimuli exceeds 75 dB SPL [11]. At lower levels of stimulus, otoacoustic emissions would barely emerge from acoustic noise in the NICU.

The whole process was automatic, driven by a hand-held, battery-driven audiological system (Elios™, Echodia). The only action undertaken by the NICU nurses was positioning of the electrodes. The fastest rate of CM sampling was one point every 10–20 s in the NICU environment. Here, for monitoring sessions scheduled to exceed 60 minutes, one CM data point was collected every 5 minutes. For monitoring sessions of 30–60 minutes, the sampling rate increased to 1/minute and for even shorter sessions, to 3/minute. In parallel, ICP was continuously monitored through an intraparenchymal probe (Codman™ microsensor, Johnson & Johnson). Data collection was stopped when nursing procedures required the monitoring equipment to be disconnected. Accidental earplug-electrode dislocation, which occurred once, led to invalid data, which was identified and discarded offline.

### Middle-ear assessment

The CM technique detects a phase rotation due to the change in stiffness of the ossicular chain induced by ICP. If middle-ear mechanics are modified by a confounding factor, e.g., a poorly ventilated or fluid-filled middle-ear cavity, the CM phase may undergo an additional rotation and the sensitivity of the CM-phase-to-ICP relationship may change. After video-otoscopic examination of the tympanic membrane, the tip of a tympanometer (Titan™, Interacoustics) was inserted. By sweeping the air pressure in the sealed ear canal, tympanometry determines whether the tympanic membrane optimally transmits sound pressure to the cochlea at atmospheric pressure. Insufficient middle-ear ventilation or middle-ear effusion is thus readily identified. As these two conditions are known to be induced by long-lasting intubation and the resulting auditory-tube dysfunction [12, 13], a subgroup of patients received tympanometry every day for 10 days while intubated (n = 6) and 6 days after discharge (n = 5), to document the time-course of possible middle-ear disruption (see Additional file 2).

### Ethics

The protocol was approved by the local Ethics Committee and National ANSM Agency, and registered in the Clinical trials register under the number NTC01685476 (protocol NIMI-NICU: Non Invasive Monitoring of the Intracranial Pressure- Neurointensive Care Unit). Informed consent was initially given by an authorized person, pending the patient's own consent, recovery permitting.

### Statistical analysis

Pearson's correlation between ICP and CM phase changes were analyzed for each monitoring session, with the correlation coefficient (*R*), the determination coefficient (*R* ^2^) and the 95% confidence interval for the correlation coefficient as outcomes. The slope of the linear regression line relating CM-phase change to ICP change was computed for each ear. Its correlation with other factors, namely age and severity indexes, was studied.

Receiver-operating characteristics (ROC) were plotted, representing sensitivity against 1 - specificity of the CM-based binary classifier, with each point corresponding to a gradually increasing discrimination threshold, defined as the CM-phase-change boundary between pass and refer diagnoses. Several ROC analyses were performed, using two different gold standards for ICP increase (against which CM phase increases were tested), 5 mm and 7.5 mmHg; and two different gold standards for ICP decrease (against which CM phase increases were tested), 5 mm and 7.5 mmHg. The optimal CM-phase-change limit was determined either according to the Youden index, or from the best trade-off between the risk of false negatives that dismiss genuinely threatening ICP increases, and of false positives that might induce an over-reaction to harmless ICP changes.

## Results

### Status of patients and outcome of their monitoring

Although all 53 patients who were tested had valid monitoring signals from the intraparenchymal ICP transducer and the CM electrodes (at least in one ear), only the data from the 24 patients who had not been intubated for more than 4 days when tested were further analyzed. These patients were aged between 19 and 76 years (mean 43, SD 16; 59% male). Seventeen patients had been referred with head trauma, three with brain hemorrhage and four with a tumor, stroke or meningitis. At referral, which occurred on average 3.9 h (SD 4.4) after symptom onset, the mean Glasgow score was 5.3 (SD 3.4), mean ICP was 18.9 mmHg (SD 11.4), with ICP over 20 mmHg in 11 patients. Severity was 54.8 (SD 14.3) on the Simplified Acute Physiology Score (SAPS-II score) and 8.2 (SD 2.2) on the Sequential Organ Failure Assessment score (SOFA score).

When their CM was monitored, all of these 24 patients had normal middle-ear function as assessed by tympanometry. Conversely, none of the excluded patients (n = 29) who had been endotracheally ventilated for more than 4 days when tested, had a normal tympanometry (negative pressure in the middle ear cavity in 8 cases, effusion in 21 cases). In the subgroup of patients who received everyday tympanometry, it was possible to check that middle-ear negative pressure, then effusion, developed from the fifth day post intubation (Additional file 2). Monitoring sessions ran from a few minutes to more than 4 h (mean 123 minutes, SD 85 minutes; total ICP monitoring data 50 h). Intermittent sound stimulations required by the CM technique did not influence the management of sedation. Figure 2 depicts representative examples of the collected ICP and CM raw data. Figure S3 in (Additional file 3) completes the picture by showing the remaining set of data.

**Fig. 1:**
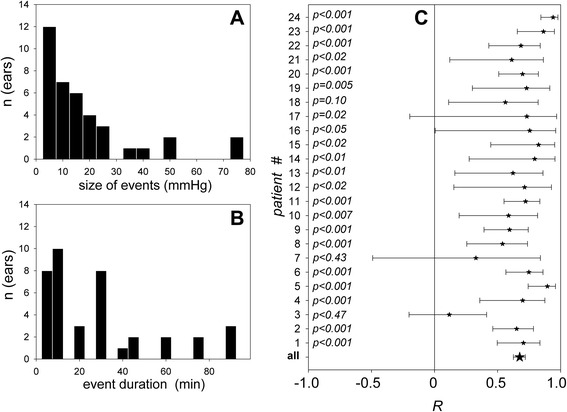
Characteristics of intracranial pressure (ICP) events and their tracking by cochlear microphonic potential (CM). **a** Size of ICP events in mmHg. **b** Duration of ICP events in minutes. **c** Pearson correlation coefficients (*R*) describing the CM-phase-to-ICP linear relationship, vertically aligned for every individual ear (*stars*), with the 95% confidence interval for each of them (*bars*)

Events, defined by changes in ICP of 3 mmHg or more, on two consecutive ICP data points at least, were observed in all patients. Overall, 38 events were registered, 18 ICP increases and 20 decreases. The amplitude of ICP changes was broadly distributed, below 20 mmHg in 29 events (Fig. 1a). A typical monitoring session included events lasting less than 40 minutes in 75% of cases (Fig. 1b), and periods of over 60 minutes with stable ICP, during which it was possible to verify whether CM phase was also stable.

**Fig. 2:**
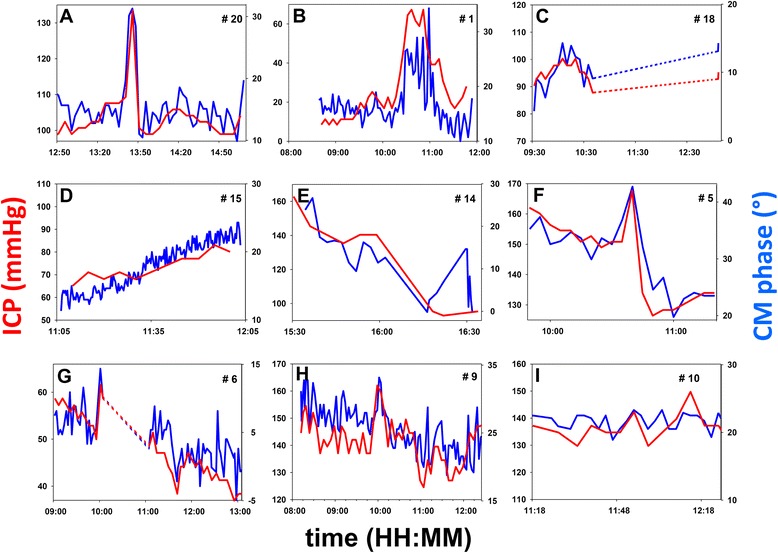
Examples of individual time courses of ICP (*red*, mmHg) and CM phase (*blue*, degrees). Vertical range: 20 mmHg for ICP in all diagrams; adjusted according to the CM-phase-to-ICP slope, for CM phase (also see Fig. 3a). In every panel labeled from **a**-**i**, patient numbers (#n, upper right corners) match those in Fig. 1c, left column. *Dashed* lines: intervals of time during which data collection was interrupted by a nursing procedure

A normal tympanogram guarantees normal sound conduction with a linear relationship between the CM phase and ICP [9], as the stiffness of the ossicular chain primarily depends on intralabyrinthine pressure that closely follows ICP [8]. This was confirmed by a correlation coefficient between 0.55 and 0.90, significantly different from 0 in 21 out of 24 patients (Fig. 1c). The inspection of individual plots (Fig. 2a-i; also see Additional file 3) confirms that the CM phase captured isolated events of a few minutes or over an hour (Fig. 2a-c) and provided a fine tracking of slow upward (Fig. 2d) or downward drifts of ICP (Fig. 2e), and of complex patterns of fluctuations (Fig. 2f-h).

**Fig. 3:**
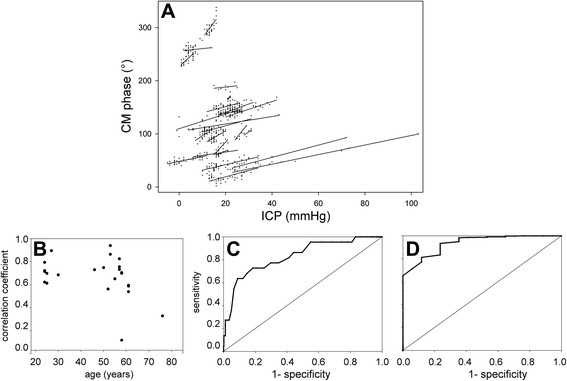
Statistical analysis of cochlear microphonic potential (*CM*) monitoring outcomes. **a** linear fits of individual CM-phase to intracranial pressure (*ICP*) relationships. **b** Pearson *R* coefficients against age. **c-d** Receiver operating characteristic plots of true positive vs fallout of binary classifications based upon a 5-mmHg increase in ICP (**c**) and a 5-mmHg decrease in ICP (**d**)

Conversely, the CM phase usually appeared stable when ICP did not change (Fig. 2a, f, i; also see Additional file 3: Figure S3I, J, L). These situations, inadequately described by the linear-correlation technique, will be quantified subsequently within this paper. In three patients, the correlation was not statistically significant (Additional file 3: Figure S3M-O). In two patients (Additional file 3: Figure S3M, O), premature interruption of data collection by a nursing procedure led to scarce ICP data points and too narrow a range of ICP variation to identify correlation, despite the close match between the CM phase and ICP. Pooled changes in the CM phase were linearly correlated with ICP changes (*R* = +0.66; *p* < 0.0001). Individual slopes of the CM-phase-to-ICP linear regression displayed some variability (Fig. 3a). Slopes were comparable whether ICP increased beyond 30 mmHg (six cases) or not, which suggests that the method can still detect ICP increases even when initial ICP is already abnormal. The average slope was 1.26 degrees of CM phase shift per mmHg increase in ICP (SD 0.74).

Age was weakly correlated with the quality of CM monitoring in relation to ICP (Fig. 3b, with borderline significance (*R* = -0.43; *p* = 0.045) possibly unduly influenced by two outliers. The slope of the CM-phase-to-ICP changes in degrees/mmHg displayed no significant correlation with age (*R* = 0.27; *p* = 0.23). Severity indexes did not explain the variability of outcomes (*p* = 0.67 and 0.31 for SAPS-II and SOFA, respectively).

### Ability of CM phase to trigger alarms as a binary classifier

A possible application of the CM-phase monitoring was assessed with the help of receiver operating characteristics (ROC), namely the ability to warn of ICP changes of +5 and -5 mmHg. For the +5 mmHg criterion (Fig. 3c; 18 events), the optimal cutoff for CM-phase shift was 10°, with sensitivity of 64% and specificity of 91%, according to Youden's index (respective 95% confidence intervals (44–84) and (89–94)). The area under the curve was 0.83. Sensitivity rose to 73% with little loss in specificity (83%) with a CM-phase shift of 7° as the cutoff.

If the alarming ICP increase was set at +7.5 mmHg instead of 5 mmHg, the ROC had an area under the curve of 0.84, with an optimal cutoff at 7° for the CM-phase shift, implying sensitivity of 83% (i.e., a 19% improvement relative to the +5 mmHg criterion), and specificity remaining good at 81%. In the mirror situation of a 5 mmHg decrease in ICP (Fig. 3d; 20 events), the area under the ROC curve was 0.93, with an optimal CM-phase shift cutoff of -5°, ensuring sensitivity of 88% and specificity of 81% according to Youden's index (respective 95% confidence intervals (73–100) and (78–84)).

## Discussion

### Ability to detect ICP trends

The close similarity between the time courses of CM-phase and invasive ICP suggests promising clinical applications. This similarity is attested by highly significant linear correlation over several hours, between the CM-phase and ICP in 21 of the 24 patients at an average rate of 1.26 degree/mmHg. To circumvent the inability of the CM method to provide absolute ICP without the help of initial invasive calibration, CM-phase changes were used as a binary classifier for tracking ICP changes (5– 7.5 mm Hg) over periods of 3–4 h, in patients with no middle-ear impairment.

The performance of the resulting classification compared favorably to those of other methods that targeted similar ICP changes [14], with sensitivity and specificity of around 80% for a dataset comprising 50 h of continuous recordings. In other words, a possibly alarming trend of ICP, defined as an increase of 5 mmHg over several minutes, was signaled by a concomitant increase in the CM-phase of 7° with 27% false negatives. In the context of objective screening for intracranial hypertension, false negatives are a more serious issue than false alarms, as in the absence of a monitoring tool all patients would have to be considered at risk.

A conservative use of ROC analysis thus chooses a more stringent CM-phase limit than that of Youden's index. False negatives decreased to 17% when an ICP increase of 7.5 mmHg was targeted. Conversely, when ICP variation remained less than 5 mmHg, the rate of false alarms that a drifting CM phase would trigger was less than 20%. The tracking of ICP decreases performed even slightly better. An important target of the present method, i.e., patients with a steady ICP increase, 5 mmHg then 7.5 and more after the accident, would thus be spotted early and efficiently enough to be directed to an appropriate emergency ward with little risk of undue alarm.

### Limitations of the monitoring method

Abnormal transmission of sound through the middle ear must be ruled out as it may influence the CM phase in an unpredictable manner. Tympanometry, which can be performed by paramedics after minimal training (and might be implemented in CM measuring devices), is recommended in the category of patients with head trauma with fractures, to rule out bilateral middle-ear involvement. In patients intubated for fewer than 5 days, middle-ear status remains normal, ensuring large CM-to-ICP correlations. In patients who stay longer in NICU, middle-ear effusion develops in relation to intubation and unconsciousness [12]. In traumatic brain injury, noninvasive ICP monitoring should no longer be needed after 4 days, as intracranial hypertension generally develops in the first 48 h after the trauma.

The CM monitoring procedure conducted with an extratympanic electrode is harmless, complying with security requirements on exposure to loud sounds, which tolerate an 8-h, 80-dB (in Europe) or 85-dB (in the USA) daily exposure. With the chosen sampling rate, suitable for detecting slow changes in ICP, patients were exposed to sound for about 20 s every 5 minutes and showed no reaction to sound presentation.

CM measurements have been routinely used for decades in audiology [15]. The present protocol is sufficiently user-friendly to be implemented by nurses whose tasks are to position an earplug in the external auditory meatus and two skin electrodes on the forehead of the patients. CM monitoring is automatic and inadvertent electrode removal is detected by the software. The cost of use is limited to that of disposable electrodes. There are similar hand-held and battery-powered versions of audiological systems that are used outside of medical facilities. Here, CM was successfully monitored in the noisy environment of NICU, suggesting that a CM-detecting device could be used aboard an emergency vehicle or even, at the site of an accident.

### Comparison to other non-invasive methods

Most non-invasive methods require initial invasive calibration [16] except transcranial Doppler ultrasound measurement of blood flows in the intra and extracranial segments of the ophthalmic artery. The estimate of absolute ICP given by this method is the pressure applied on the eyeball, such that blood flow in both segments of the ophthalmic artery is equalized [17, 18]. For ICPs below 40 mmHg, the reported standard deviation of the error is small, 6.2 mmHg. Yet, the procedure is too intrusive for continuous monitoring and requires advanced technical skills. The method of Kashif et al. [14] estimates ICP every minute, with little bias and a satisfactory standard deviation of the error (7.6 mmHg) for ICPs up to 100 mmHg. However, it requires a model simulating brain properties to be fed by minimally invasive clinical data, arterial blood pressure and cerebral blood flow derived from transcranial ultrasound.

Invasive ICP measurements are not exempt from technical problems. The baseline drift of ICP microtransducers reaches up to 7.3 mmHg so that the standard deviation of the error may vary from 3.4 to 8.5 mmHg [19]. External ventricular drainage may be obstructed or disabled by intraventricular hemorrhage or ventricular compression by brain edema. In addition, ICP values provided by intraparenchymal microtransducers may depend on the site of placement [20]. When placed in one hemisphere they may not accurately reflect the overall brain pressure if contusions build up secondarily in the other hemisphere. In these circumstances, the CM method based on the equilibration between intralabyrinthine and cerebrospinal fluid pressures may provide complementary information on global brain pressure after initial calibration.

### Clinical targets of CM monitoring

The patients in this study were selected because the gold standard of invasive ICP monitoring was required to assess the CM technique. They do not constitute the realistic target population for noninvasive ICP monitoring. The present preliminary results suggest a potential interest for the CM technique to be used whenever invasive monitoring of ICP is unavailable or contraindicated, keeping in mind the two limitations of CM, i.e., the need for calibration to access absolute ICP and the risk of false negatives due to the imperfect sensitivity.

With a strategy based upon the detection of an increase in ICP of 7.5 mmHg, the same order of magnitude as the standard deviation of the error of the best noninvasive absolute methods published so far, CM monitoring would spot significant ICP trends in patients starting from a clinically satisfactory status, suggesting a likely normal initial ICP. At the scene of an accident, particularly in a remote area far from acute-care hospitals, a noninvasive method of detecting an increase in ICP could be crucial in patients initially categorized as having “mild brain injury” before the onset of consciousness disorders. Mild traumatic brain injuries are usually managed in a general hospital if early computed tomography is normal. A significant change in CM could signal the formation of an intracranial hematoma and, together with the clinical methods currently in use, help assess whether transfer to a neurosurgical center is appropriate.

Management of cerebrovascular accidents provides similar indications. In patients with ambiguous clinical assessment, our method could contribute to early detection of brain edema when ICP starts increasing. Patients under the influence of alcohol or treated with platelet anti-aggregants or anticoagulants require special attention because they are susceptible to worsening and difficult to monitor clinically. In these cases, non-invasive monitoring of ICP could help decide whether transfer to an NICU is required. Other indications include the pediatric population whose lack of compliance might make medical surveillance difficult, and the follow up of patients referred to emergency wards for severe headache on suspicion of intracranial hyperpressure, when invasive methods cannot be performed. In addition to primarily being used for screening in departments without the possibility of invasive monitoring, CM may serve as an outpatient tool in patients with long-lasting ICP problems, after initial calibration by an invasive ICP measurement.

## Conclusions

In conclusion, the portability and cost-effectiveness of the CM method, usable by paramedics in emergency vehicles or in patients’ homes, makes it promising for use in patients who cannot currently benefit from ICP monitoring.

## Additional files

Additional file 1: Figure S1. CM measuring equipment; the noninvasive setup that detects ICP changes in a subject monitored in a non-medical environment (PDF 25 kb)
Additional file 2 Effects of long-lasting endotracheal ventilation on ICP variability and on middle-ear status. **Table S1.** The variability of ICP decreases with increasing duration of NICU stay. Brief description of the middle-ear monitoring protocol. **Figure S2.** Percentage of ears with normal and abnormal middle-ear function as a function of the number of days after referral and intubation, then after extubation (PDF 172 kb)
Additional file 3: Figure S3. Time course of ICP and the CM phase in all the patients who are not represented in Fig. 2 (PDF 284 kb)

## Abbreviations

CM: Cochlear microphonic potential
ICP: Intracranial pressure
NICU: Neuro-intensive care unit
ROC: Receiver-operating characteristics
SAPS-II: Simplified Acute Physiology Score
SOFA: Sequential Organ Failure Assessment
SPL: sound pressure level

## References

[1] Miller JD, Stanek A, Langfitt TW (1972). Concepts of cerebral perfusion pressure and vascular compression during intracranial hypertension. Prog Brain Res..

[2] Czosnyka M, Pickard JD (2004). Monitoring and interpretation of intracranial pressure. J Neurol Neurosurg Psychiatry.

[3] Guillaume J, Janny P (1951). Continuous intracranial manometry; importance of the method and first results. Rev Neurol (Paris).

[4] Chesnut R, Videtta W, Vespa P, Le Roux P (2014). Participants in the International Multidisciplinary Consensus Conference on Multimodality M. Intracranial pressure monitoring: fundamental considerations and rationale for monitoring. Neurocrit Care.

[5] Le Roux P, Menon DK, Citerio G, Vespa P, Bader MK, Brophy G, Diringer MN, Stocchetti N, Videtta W, Armonda R (2014). The International Multidisciplinary Consensus Conference on Multimodality Monitoring in Neurocritical Care: a list of recommendations and additional conclusions: a statement for healthcare professionals from the Neurocritical Care Society and the European Society of Intensive Care Medicine. Neurocrit Care..

[6] Buki B, Avan P, Lemaire JJ, Dordain M, Chazal J, Ribari O (1996). Otoacoustic emissions: a new tool for monitoring intracranial pressure changes through stapes displacements. Hear Res.

[7] Avan P, Buki B, Maat B, Dordain M, Wit HP (2000). Middle ear influence on otoacoustic emissions. I: noninvasive investigation of the human transmission apparatus and comparison with model results. Hear Res.

[8] Traboulsi R, Avan P (2007). Transmission of infrasonic pressure waves from cerebrospinal to intralabyrinthine fluids through the human cochlear aqueduct: Non-invasive measurements with otoacoustic emissions. Hear Res.

[9] Buki B, Giraudet F, Avan P (2009). Non-invasive measurements of intralabyrinthine pressure changes by electrocochleography and otoacoustic emissions. Hear Res.

[10] Minto CF, Schnider TW, Gregg KM, Henthorn TK, Shafer SL (2003). Using the time of maximum effect site concentration to combine pharmacokinetics and pharmacodynamics. Anesthesiology.

[11] Avan P, Buki B, Petit C (2013). Auditory distortions: origins and functions. Physiol Rev.

[12] Hamill-Ruth RJ, Ruth RA (2003). Evaluation of audiologic impairment in critically ill patients: results of a screening protocol. Crit Care Med.

[13] Cavaliere F, Masieri S, Liberini L, Proietti R, Magalini SI (1992). Tympanometry for middle-ear effusion in unconscious ICU patients. Eur J Anaesthesiol.

[14] Kashif FM, Verghese GC, Novak V, Czosnyka M, Heldt T (2012). Model-based noninvasive estimation of intracranial pressure from cerebral blood flow velocity and arterial pressure. Sci Transl Med.

[15] Probst R (1983). Electrocochleography: using extratympanic or transtympanic methods?. ORL J Otorhinolaryngol Relat Spec.

[16] Popovic D, Khoo M, Lee S (2009). Noninvasive monitoring of intracranial pressure. Recent Patents Biomed Eng.

[17] Firsching R, Muller C, Pauli SU, Voellger B, Rohl FW, Behrens-Baumann W (2011). Noninvasive assessment of intracranial pressure with venous ophthalmodynamometry. Clinical article. J Neurosurg..

[18] Ragauskas A, Matijosaitis V, Zakelis R, Petrikonis K, Rastenyte D, Piper I, Daubaris G (2012). Clinical assessment of noninvasive intracranial pressure absolute value measurement method. Neurology.

[19] Gelabert-Gonzalez M, Ginesta-Galan V, Sernamito-Garcia R, Allut AG, Bandin-Dieguez J, Rumbo RM (2006). The Camino intracranial pressure device in clinical practice. Assessment in a 1000 cases. Acta Neurochir (Wien).

[20] Raboel PH, Bartek J, Andresen M, Bellander BM, Romner B (2012). Intracranial pressure monitoring: invasive versus non-invasive methods - a review. Crit Care Res Pract..

