# Supplementary material for: Noninvasive detection of alarming intracranial pressure changes by auditory monitoring in early management of brain injury: a prospective invasive versus noninvasive study
Source: Crit Care. 2017 Feb 21;21:35. doi: 10.1186/s13054-017-1616-2 (PMC5319090; doi:10.1186/s13054-017-1616-2)
Supplement: Additional file 1: Figure S1. — CM measuring equipment; the noninvasive setup that detects ICP changes in a subject monitored in a non-medical environment (PDF 25 kb) [file 13054_2017_1616_MOESM1_ESM.pdf]

Field deployability and ease of use of the CM-monitoring device

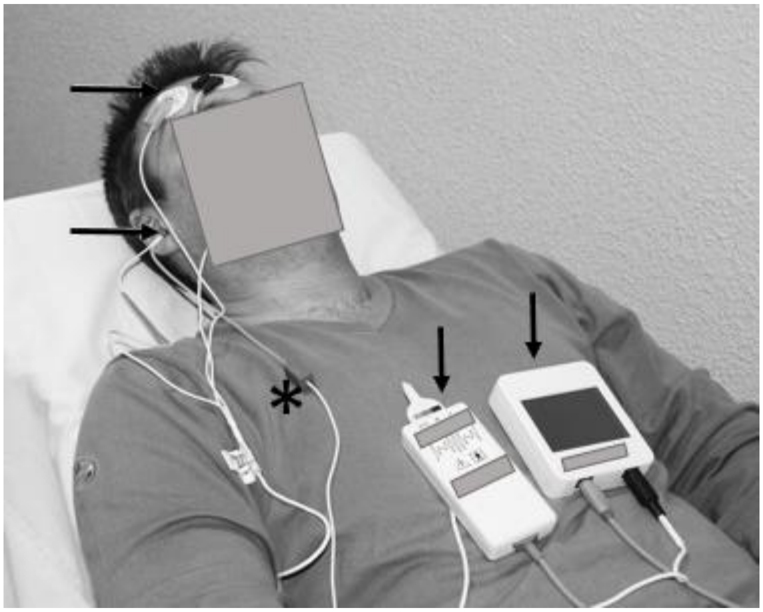

Fig. S1. CM-measuring device in situ. Picture of the setup in a non-medical environment (vertical arrows: CM-device, preamplifier; horizontal arrows: electrodes; *: earphone). The earphone is connected to the ear by a 30-cm long plastic tubing. Before starting CM data collection, this tubing can be clamped to attenuate the sound stimulus, which allows the operator to check the absence of electromagnetic artifact emitted by the earphone.
