# Supplementary material for: Noninvasive detection of alarming intracranial pressure changes by auditory monitoring in early management of brain injury: a prospective invasive versus noninvasive study
Source: Crit Care. 2017 Feb 21;21:35. doi: 10.1186/s13054-017-1616-2 (PMC5319090; doi:10.1186/s13054-017-1616-2)
Supplement: Additional file 2 — Effects of long-lasting endotracheal ventilation on ICP variability and on middle-ear status. Table S1. The variability of ICP decreases with increasing duration of NICU stay. Brief description of the middle-ear monitoring protocol. Figure S2. Percentage of ears with normal and abnormal middle-ear function as a function of the number of days after referral and intubation, then after extubation (PDF 172 kb) [file 13054_2017_1616_MOESM2_ESM.pdf]

In the 29 patients who had stayed more than 4 days in NICU before CM monitoring could be organized, the CM amplitudes and signal-to-noise ratios remained larger than 10 dB. However, they were excluded from further analysis because their case raised two problems. In the first place, their ICP had become properly controlled by a medical treatment started at least 4 days earlier. ICP fluctuations (ΔICP in Table S1 below) from one measurement point to the next were computed and their standard deviations were compared among groups of patients using the test of Bartlett (Table S1). ICP variability, about half that of patients monitored earlier, was significantly decreased ($p<0.001$), un unfavorable factor for the search for correlations between CM phase and ΔICP to be valid.

Intubation time	ΔCM phase (degrees)	ΔICP (mmHg)
More than 6 days (n = 21)	13.3	2.19
Between 4 and 6 days (n = 8)	12.7	1.94
Less than 4 days (n = 24, included in the main analysis)	8.48	3.56
p for Bartlett's Test	<0.001	<0.001

Table S1. Standard deviations of CM and ICP fluctuations among groups of patients.

A second issue is that negative pressure build-up in the middle ear cavities and fluid effusion are thought to occur in many endotracheally-ventilated patients after several days, due to the insufficient ventilation of their middle-ear cavities. The consequences of changed middle-ear mechanics (increased tympanic-membrane stiffness and altered middle-ear inertial properties) on how CM phase reacts to a change in ICP are difficult to model quantitatively as increased middle-ear stiffness tends to shift resonance frequencies upward, whereas increased inertia induces a downward shift. The likely varying trade-off between the two trends makes it impossible to predict whether the ICP-to-CM phase relationship would become shallower or steeper. The increased variability of CM phase in patients intubated more than 4 days (Table S1, middle column; $p<0.001$) reflects this uncertainty.

The progressive change in middle-ear status with prolonged intubation was carefully demonstrated here in a subsample of 6 patients whose middle-ear status was measured bilaterally by tympanometry every day for 10 days, after referral and tracheal intubation.

On the first 3 days, all middle ears were normal (n = 12 ears; Fig.S2 left). From day 5 on, the proportion of middle ears with fluid effusion increased from 25% to 100%. In between, a few patients displayed negative pressures in their middle-ears as an intermediate condition. Video-otoscopy confirmed the presence of middle-ear effusion in all patients after 7 days. The follow-up of middle-ear status in patients (n = 5) tested bilaterally after extubation, every day during 6 days, showed the progressive reversibility of tympanogram anomalies provoked by intubation (Fig.S2 right).

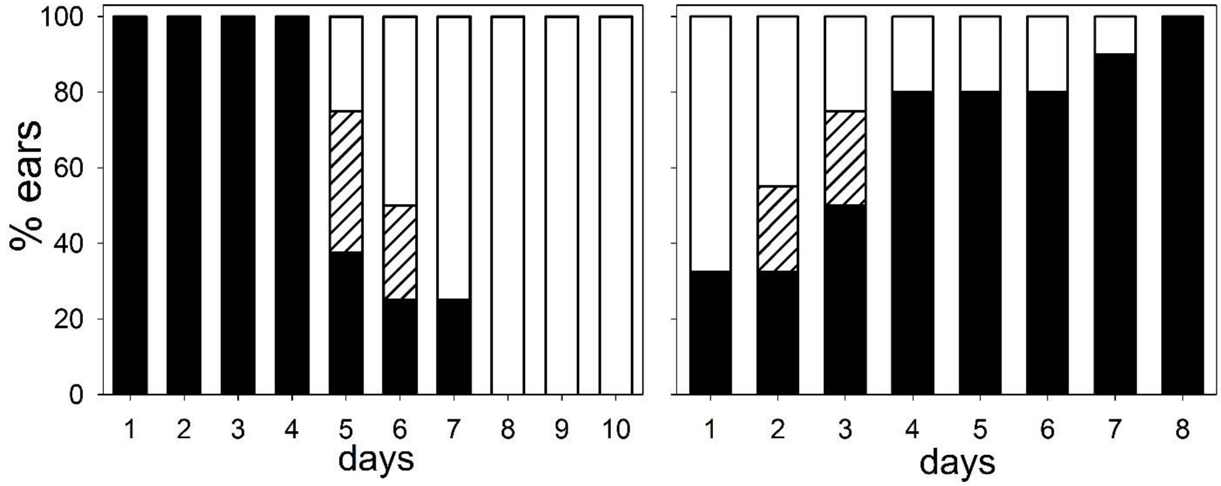

Fig.S2. Daily follow-up by tympanometry. (left) horizontal axis, number of days after intubation; vertical axis, number of ears with normal middle-ear cavity (solid), negative pressure build-up (hatched) and fluid effusion (open box). (right) horizontal axis, number of days after extubation.
