# Supplementary material for: Noninvasive detection of alarming intracranial pressure changes by auditory monitoring in early management of brain injury: a prospective invasive versus noninvasive study
Source: Crit Care. 2017 Feb 21;21:35. doi: 10.1186/s13054-017-1616-2 (PMC5319090; doi:10.1186/s13054-017-1616-2)
Supplement: Additional file 3: Figure S3. — Time course of ICP and the CM phase in all the patients who are not represented in Fig. 2 (PDF 284 kb) [file 13054_2017_1616_MOESM3_ESM.pdf]

Monitoring of ICP in patients who stayed less than 4 days in NICU

The following panels (**A-O**) display the time courses of ICP and CM phase in all the patients not shown in Fig.2 in the main text.

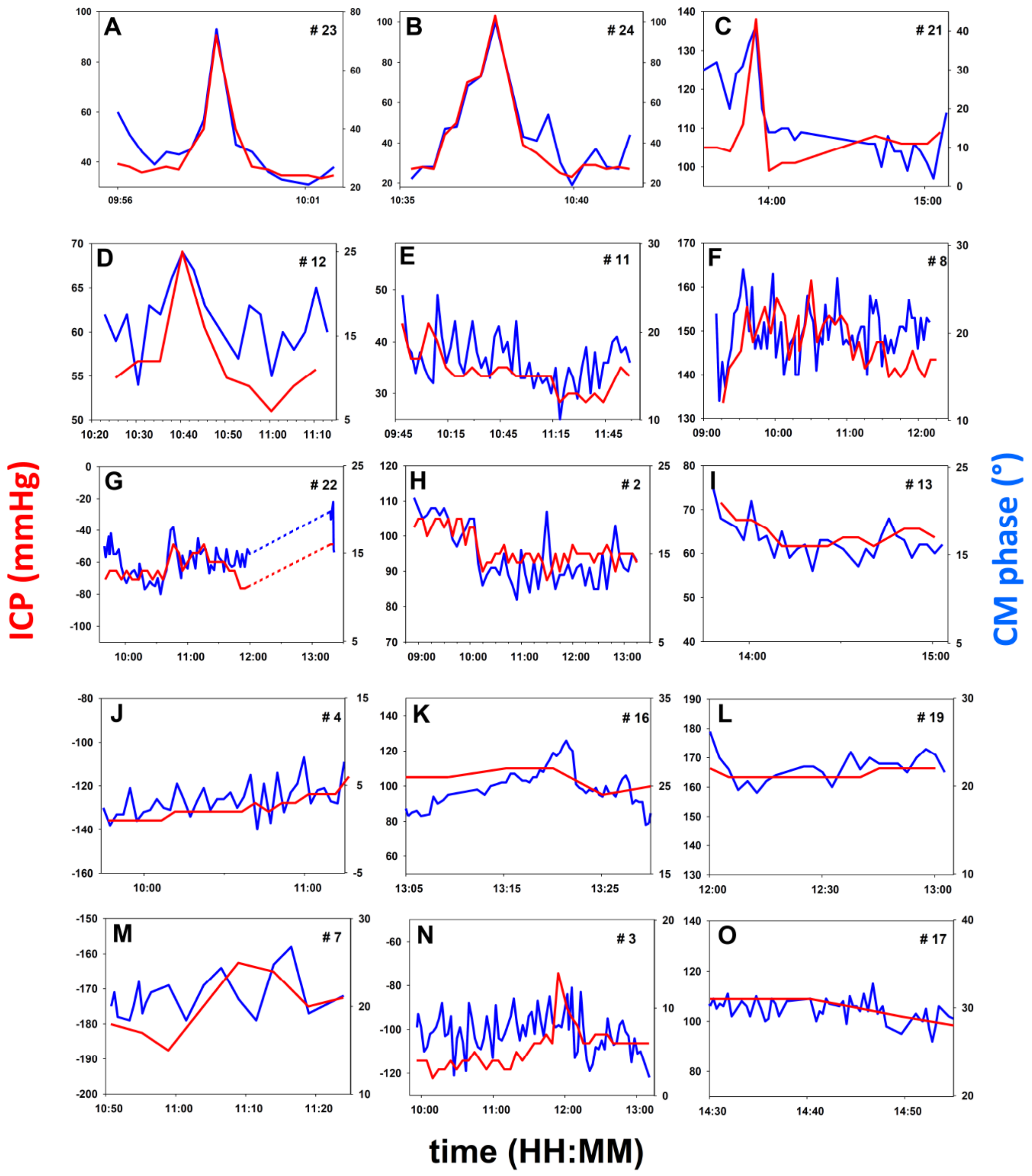

Fig.S3: ICP (red, in mmHg) and CM phase (blue, in degrees). Patient numbers (#n) match those in fig.1**C**, left column (main text). Diagrams A to L are sorted according to the size of ICP changes over the time course of monitoring, in descending order. In the lowest row (M-O), CM-to-ICP relationships do not reach significance.
